# Identification of novel plasma glycosylation-associated markers of aging

**DOI:** 10.18632/oncotarget.7059

**Published:** 2016-01-28

**Authors:** Mariangela Catera, Vincenzo Borelli, Nadia Malagolini, Mariella Chiricolo, Giulia Venturi, Celso A. Reis, Hugo Osorio, Provvidenza M. Abruzzo, Miriam Capri, Daniela Monti, Rita Ostan, Claudio Franceschi, Fabio Dall'Olio

**Affiliations:** ^1^ Dipartimento di Medicina Specialistica Diagnostica e Sperimentale (DIMES) University of Bologna, Bologna, Italy; ^2^ Instituto de Investigação e Inovação em Saúde (Institute for Research and Innovation in Health), University of Porto, and Institute of Molecular Pathology and Immunology of The University of Porto IPATIMUP), Porto, Portugal; ^3^ Institute of Biomedical Sciences Abel Salazar (ICBAS), University of Porto, Porto, Portugal; ^4^ Faculty of Medicine of The University of Porto, Al. Prof. Hernâni Monteiro, Porto, Portugal; ^5^ Dipartimento di Scienze Biomediche, Sperimentali e Cliniche “Mario Serio”, University of Florence, Florence, Italy

**Keywords:** antibody glycosylation, inflammaging, plasma galactosyltransferases, plasma sialyltransferases, soluble glycosyltransferases, Gerotarget

## Abstract

The pro- or anti-inflammatory activities of immunoglobulins G (IgGs) are controlled by the structure of the glycan N-linked to Asn_297_ of their heavy chain. The age-associated low grade inflammation (inflammaging) is associated with increased plasmatic levels of agalactosylated IgGs terminating with N-acetylglucosamine (IgG-G0) whose biogenesis has not been fully explained. Although the biosynthesis of glycans is in general mediated by glycosyltransferases associated with internal cell membranes, the extracellular glycosylation of circulating glycoproteins mediated by plasmatic glycosyltransferases has been recently demonstrated. In this study we have investigated the relationship between plasmatic glycosyltransferases, IgG glycosylation and inflammatory and aging markers. In cohorts of individuals ranging from infancy to centenarians we determined the activity of plasmatic β4 galactosyltransferase(s) (B4GALTs) and of α2,6-sialyltransferase ST6GAL1, the glycosylation of IgG, the GlycoAge test (a glycosylation-based marker of aging) and the plasma level of inflammatory and liver damage markers. Our results show that: 1) plasmatic B4GALTs activity is a new marker of aging, showing a linear increase throughout the whole age range. 2) plasmatic ST6GAL1 was high only in children and in people above 80, showing a quadratic relationship with age. 3) Neither plasmatic glycosyltransferase correlated with markers of liver damage. 4) plasmatic ST6GAL1 showed a positive association with acute phase proteins in offspring of short lived parents, but not in centenarians or in their offspring. 5) Although the glycosylation of IgGs was not correlated with the level of the two plasmatic glycosyltransferases, it showed progressive age-associated changes consistent with a shift toward a pro-inflammatory glycotype.

## INTRODUCTION

Aging has been recently proposed as a continuation of developmental growth [1] and is characterized by a chronic, low grade, asymptomatic process, defined as “inflammaging” [2-5], whose individual level is related to biological age and risk of age-related diseases. Thus, the identification of new biomarkers of biological aging is highly relevant to profile the general health conditions of elderly population and to assess the impact of different life styles on aging.

Glycosylation, one of the most frequent postranslational modifications of proteins, undergoes profound changes associated with aging, cancer and inflammatory conditions [6-8]. Protein-linked sugar chains often modulate the biological functions of the glycoprotein. A good example is provided by the asparagine-linked (N-linked) sugar chain attached to Asn_297_ of IgG which, according to the presence or absence of specific terminal sugars, modulate the pro- or anti inflammatory properties of the antibody [9, 10]. It is known since the ’80s that the blood of rheumatoid arthritis patients contains IgG molecules with sugar chains lacking sialic acid and galactose and terminating with N-acetylglucosamine (GlcNAc), which are referred to as IgG-G0 [11]. Increased presence of IgG-G0 was described subsequently in blood of aging people [12] and in a variety of other inflammatory conditions (reviewed in [8]). The GlycoAge test is a widely recognized glycosidic marker of biological age [13-16], based on the relative abundance, in plasmatic glycoproteins, of two N-glycan species (an agalactosylated core fucosylated biantennary and a bigalactosylated, core fucosylated biantennary), as detected by DNA sequencer-aided fluorophore assisted carbohydrate electrophoresis (DSA-FACE) [17].

The biosynthesis of the sugar chains is mediated by glycosyltransferases, a class of enzymes which usually transfer single monosaccharides from sugar-nucleotide donors to sugar chains. Glycosyltransferases are classified according to the monosaccharide they transfer (galactosyltransferases, sialyltransferases etc.) and are present in cell bound forms, mainly associated with internal membranes, but also in soluble forms in body fluids [18-20]. The biogenesis of IgG-G0 in aging has never been investigated, while conflicting results have been reported on the biogenesis of IgG-G0 in rheumatoid arthritis (RA) patients. In fact, some studies reported unchanged expression of galactosyltransferase in B lymphocytes of RA patients [21-24], while others reported decreased expression [25-28]. It has long been thought that soluble plasmatic glycosyltransferases could not transfer sugars to circulating glycoproteins because of the insufficient concentration of the appropriate nucleotide-sugar donors. However, this view has recently been challenged by the finding that extracellular glycosylation can take place using the sugar nucleotide donors contained in platelets [29, 30]. Thus, the possibility that the glycosylation of plasmatic glycoproteins, including IgG, might be mediated by soluble glycosyltransferases should be taken into consideration [31]. In the present study we have measured in plasma of a cohort of individuals ranging from infancy to centenarians, the activity of α2,6-sialyltransferase ST6GAL1 and of β4-galactosyltransferases (B4GALTs), enzymes which might be crucial for the level of IgG-G0 and of anti-inflammatory α2,6-sialylated IgG, determined the GlycoAge test and studied the glycosylation of IgG. Finally, the two plasmatic glycosyltransferase activities were correlated with metabolic and inflammatory markers in centenarians, persons with a genetic background putatively bound to extreme longevity, in a group of centenarian's offspring, characterized by higher probability to become long-lived, and in the offspring of non-long lived parents.

## RESULTS

### Plasmatic ST6GAL1 and B4GALTs activity in subjects of different ages

**Figure 1:**
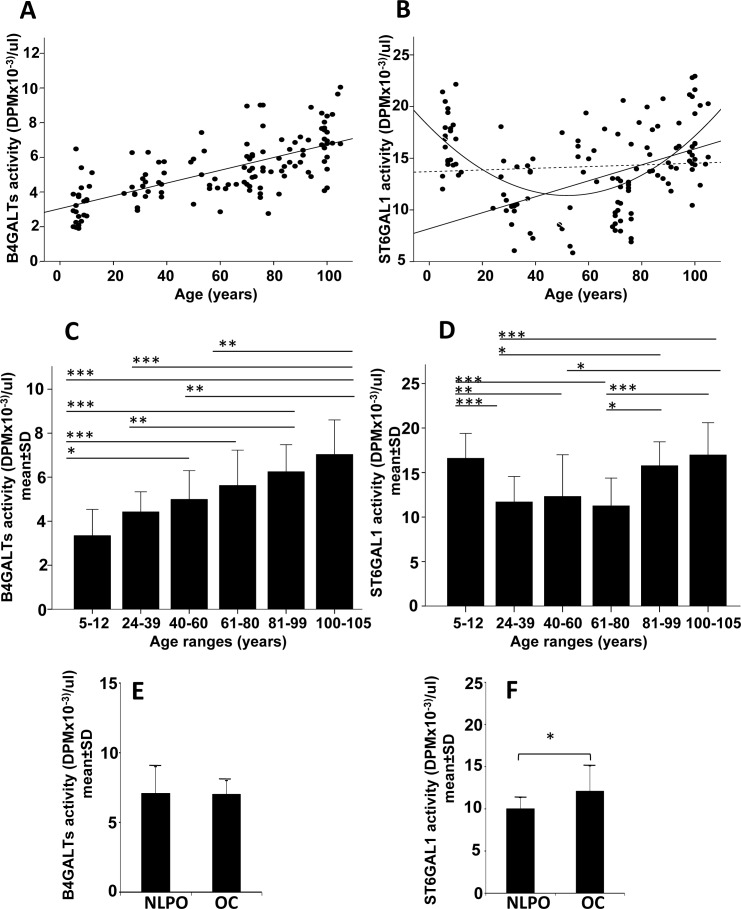
Plasmatic B4GALTs and ST6GAL1 activities in dependence of age **A.**, **C.**, **E.** plasmatic B4GALTs; **B.**, **D.**, **F.** plasmatic ST6GAL1. A and B: regression analysis of enzyme activities in subjects of different ages; C and D: activity of the two enzymes in different age groups; E and F: activity of the two enzymes in offspring of centenarians (OC) or in age- matched offspring of non-long lived parents (NLPO). A: R^2^ linear (*N* = 125, all subjects) = 0.466; *p* < 0.00001. B: R^2^ quadratic (*N* = 125, all subjects) = 0.292; *p* < 0.00001; R^2^ linear (*N* = 125, all subjects, dashed line) = 0.0001 *p* N.S.; R^2^ linear (*N* = 102, only adults) = 0.216; p < 0.00001. Comparison of the two glycosyltransferase activities in different age-groups (C and D) and in NLPO and OC (E and F). C-F: *p < 0.05; **p < 0.01; ***p < 0.001 according to Kruskal-Wallis non parametric test.

The level of ST6GAL1 and B4GALTs was measured in the plasma of individuals of both gender belonging to six age groups: children, young, middle aged, old, oldest old and centenarians. Details on the subjects are described in Materials and Methods. The acceptor used for sialyltransferase activity ensures good specificity for ST6GAL1, while that used for galactosyltransferase(s) can putatively detect the products of different B4GALT genes, although B4GALT1 is probably the major. For these reasons the two activities will be thereafter referred to as ST6GAL1 and B4GALTs. Correlation analysis showed a highly significant linear positive relationship (R^2^ = 0.466; *p* < 0.00001) of plasmatic B4GALTs with age from infancy to centenarians (Figure 1A). On the contrary, sialyltransferase ST6GAL1 displayed a highly significant quadratic relationship (R^2^ = 0.292, *p* < 0.00001) from infancy to centenarians, while an age-dependent significant linear relationship (R^2^ = 0.216, *p* < 0.00001) was observed only from young to centenarians (Figure 1B). No significant linear relationship was observed when all subjects were considered. When the mean galactosyltransferase activity of the six age groups was compared, significant differences were observed among the different classes (Figure 1C). By contrast, we observed significant differences between the ST6GAL1 activity of intermediate age groups and those of children and those above the age of 80 (Figure 1D).

To investigate whether the increased activity of plasmatic B4GALTs and ST6GAL1 was due to a particular genetic background associated with extreme longevity, we measured the two activities in 19 offspring of centenarians (OC) and 19 sex and age-matched non long-lived parent offspring (NLPO). Details on both cohorts are reported in Materials and Methods. While the activity of B4GALTs was the same in the two groups (Figure 1E), ST6GAL1 displayed a statistically significant (*p* < 0.05) 20% higher activity in OC, compared with NLPO (Figure 1F).

### Correlation between plasmatic glycosyltransferase activities and the GlycoAge test

**Figure 2:**
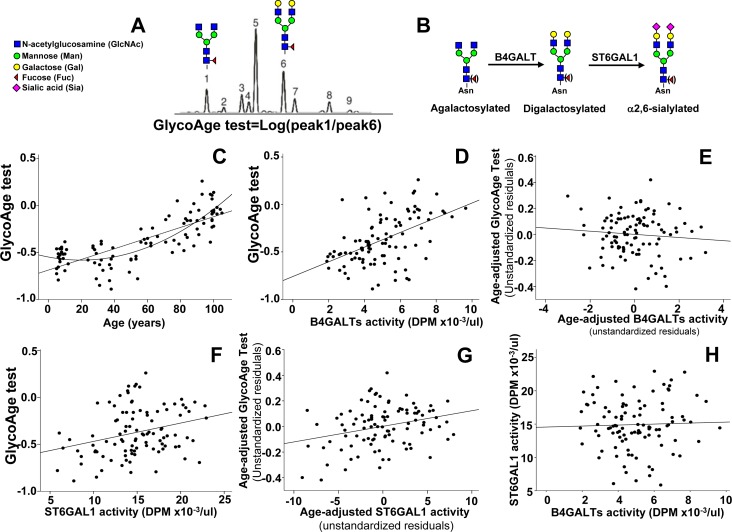
GlycoAge test and plasmatic glycosyltransferases **A.** example of DSA-FACE analysis of glycans released by a plasma sample. The Log of the ratio of the relative abundance of peaks 1 and 6, whose structure is reported above, is the GlycoAge test. Peak 1 is the agalactosylated glycan also referred to as NGA2F present in IgG-G0. Peak 6 is its bigalactosylated counterpart also referred to as NA2F. **B.** Role of B4GALTs and of ST6GAL1 in the biosynthesis of N-glycans. Core-linked fucose is in parenthesis to indicate that its presence is not necessary for the activity of the two glycosyltransferases. **C.** correlation between the GlycoAge test and age in individuals from infancy to centenarians. Both the linear and quadratic relationships were significant (linear: R^2^ = 0.608; *p* <= 0.00001; quadratic: R^2^ = 0.689, *p* < 0.00001), but the latter fit better. **D.** the highly significant relationship between the GlycoAge test and B4GALTs activity (R^2^ = 0.263; *p* = 0.00001), was lost after age-adjustment **E.**. **F.** the significant relationship between the GlycoAge test and plasmatic ST6GAL1 activity (R^2^ = 0.085; *p* = 0.0032) was maintained after age adjustment (R^2^ = 0.083; p = 0.0036) **G. H.** no significant relationship existed between plasmatic ST6GAL1 and B4GALTs activities (R^2^ = 0.003; p 0.561).

Analysis by DSA-FACE of the N-glycans released from plasmatic glycoproteins provides a limited number of peaks, corresponding to N-linked chains of defined structures (Figure 2A and 2B). In particular, the Log of the ratio between peak 1 and peak 6, corresponding to core-fucosylated diantennary N-linked chains with two terminal galactose residues (peak 6 NA2F) or terminating with GlcNAc (peak 1 NGA2F) is the GlycoAge test. In Figure 2C is shown that the relationship between age and the GlycoAge test was linear in the range 24 years to centenarians. However, we show here for the first time that if children were included in the analysis, the GlycoAge test displayed a quadratic relationship with age, instead of the usual linear relationship. The GlycoAge test and the activities of plasmatic B4GALTs and ST6GAL1 all displayed a clear dependence on calendar age, although according to different patterns. To establish whether the three markers of aging were regulated in parallel, we performed correlation analysis of the three parameters after age-adjustments. A highly significant relationship existed between plasmatic B4GALTs activity and the GlycoAge test (*p* < 0.00001, Figure 2D). However, after age adjustment the significant relationship was lost (Figure 2E, unstandardized residuals). This means that individuals of the same age group showing high GlycoAge test do not necessarily display a tendency toward high B4GALTs activity and vice versa. The relationship between GlycoAge test and ST6GAL1 activity was also significant (*p* = 0.0032, Figure 2F) and displayed little changes after age adjustment (Figure 2G). Also the relationship between ST6GAL1 and B4GALTs was not significant (Figure 2H). These data suggested that the physio-pathological mechanisms at the basis of the GlycoAge test are probably similar to those at the basis of plasmatic ST6GAL1 but different from those at the basis of plasmatic B4GALTs.

### Relationship of plasmatic glycosyltransferases with inflammatory and liver damage markers

The association of the two plasmatic glycosyltransferase activities with blood markers of inflammation/coagulation and liver damage was investigated in centenarians, OC and NLPO (Table 1). In centenarians only, it was observed a significant but negative association between GPT transaminase and B4GALTs activity. The lack of positive relationship between markers of liver damage and either plasmatic glycosyltransferase activity suggested that the release of the two enzymes was not a consequence of liver cell injuries. Only in NLPO, we observed a close positive association between plasmatic ST6GAL1 and two acute phase proteins (C reactive protein and serum amyloid A) and TGF-1β, but not with inflammatory cytokines, such as IL-6 and TNF-α-resistin etc.

**Table 1:** Correlation between plasmatic B4GALT or ST6GAL1 activities and plasma markers in centenarians (CENT), offspring of centenarians (OC) and non-long lived parents offspring (NLPO)

B4GALT						
	CENT		OC		NLPO	
	*p*	ρ	*p*	ρ	*p*	ρ
**Inflammation/coagulation markers**						
IL-6	0.990	0.003	0.842	0.051	0.875	−0.038
IL-10	0.990	−0.003	0.349	0.234	0.977	−0.007
TNF-α	0.734	0.081	0.126	0.375	0.750	−0.076
TGF-1β	0.545	−0.144	0.409	0.207	0.779	−0.067
C-reactive protein	0.357	0.218	0.990	0.030	0.111	−0.367
Serum amyloid-A	0.067	0.418	0.742	0.084	0.532	−0.152
Resistin	0.531	0.149	0.458	−0.187	0.691	0.095
Von Willebrandt Factor	0.373	−0217	0.358	0.230	0.753	0.082
**Liver damage markers**						
GOT transaminase	0.094	−0.385	0.099	0.401	0.877	−0.037
GPT transaminase	**0.009**	**-0.569**	0.239	0.293	0.182	−0.311
GGT	0.940	0.180	0.452	0.189	0.536	−0.147

ST6GAL1						
	CENT		OC		NLPO	
	*p*	ρ	*p*	ρ	*p*	ρ
**Inflammation/coagulation markers**						
IL-6	0.980	−0.060	0.754	0.080	0.340	0.225
IL-10	0.613	−0.210	0.598	−0.133	0.462	0.175
TNF-α	0.441	−0.183	0.185	−0.327	0.352	0.220
TGF-1β	0.712	0.088	0.705	0.096	**0.039**	**0.464**
C-reactive protein	0.920	0.024	0.104	0.395	**0.030**	**0.484**
Serum amyloid-A	0.640	0.111	0.081	0.422	**0.016**	**0.529**
Resistin	0.188	−0.307	0.855	0.046	0.552	0.141
Von Willebrandt Factor	0.614	−0.124	0.172	0.337	0.322	−0.256
**Liver damage markers**						
GOT transaminase	0.902	−0.029	0.665	−0.111	0.597	0.126
GPT transaminase	0.831	0.051	0.433	−0.197	0.175	0.316
GGT	0.401	−0.199	0.219	−0.304	0.470	0.448

The correlation between the plasmatic activity of B4GALTs or ST6GAL1 with several markers of inflammation/coagulation or liver damage was determined according to Spearman in the three cohorts of CENT, OC and NLPO. Statistically significant relationships are highlighted in bold.

### Glycosylation of IgG heavy chains

**Figure 3:**
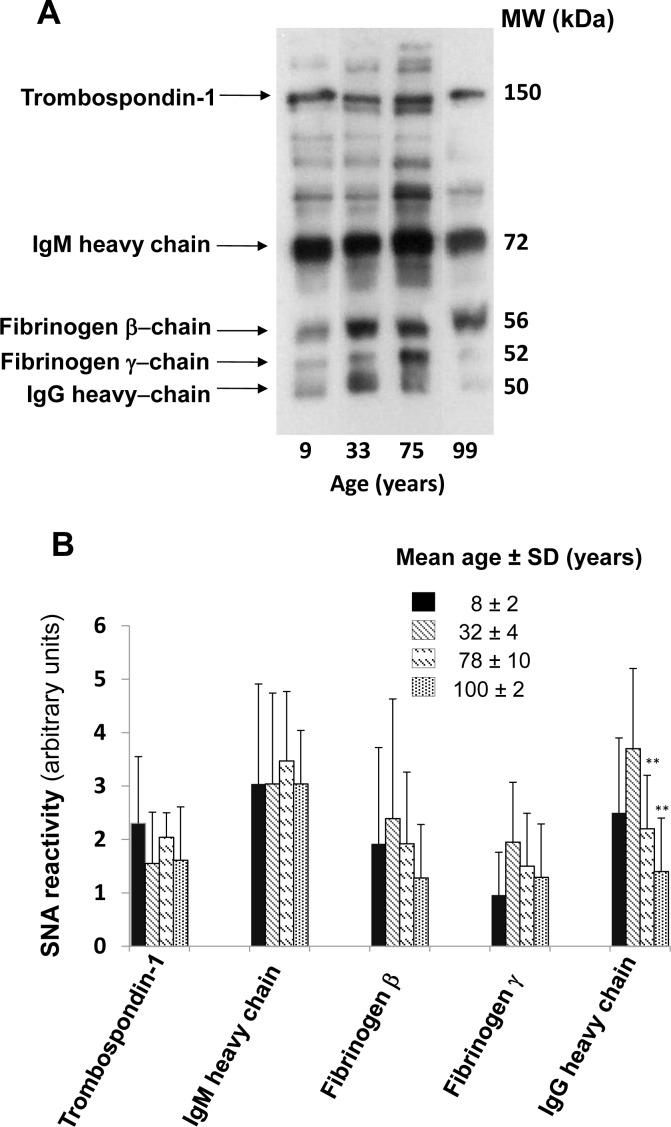
Age-dependent α2,6-sialylation of major plasmatic glycoproteins **A.** One hundred fold diluted human plasma was analyzed by SNA lectin blot. The identity of the major SNA reactive bands, identified by MALDI-TOF/TOF mass spectrometry is indicated on the left. **B.** Quantification of SNA-reactive bands in the indicated four age groups. The mean ± SD of the SNA reactivity of the five identified glycoproteins in the four age groups is reported. Only the SNA-reactivity of IgG heavy chains showed a statistically significant age-dependent decrease (Student's *t* test for independent samples).

**Figure 4:**
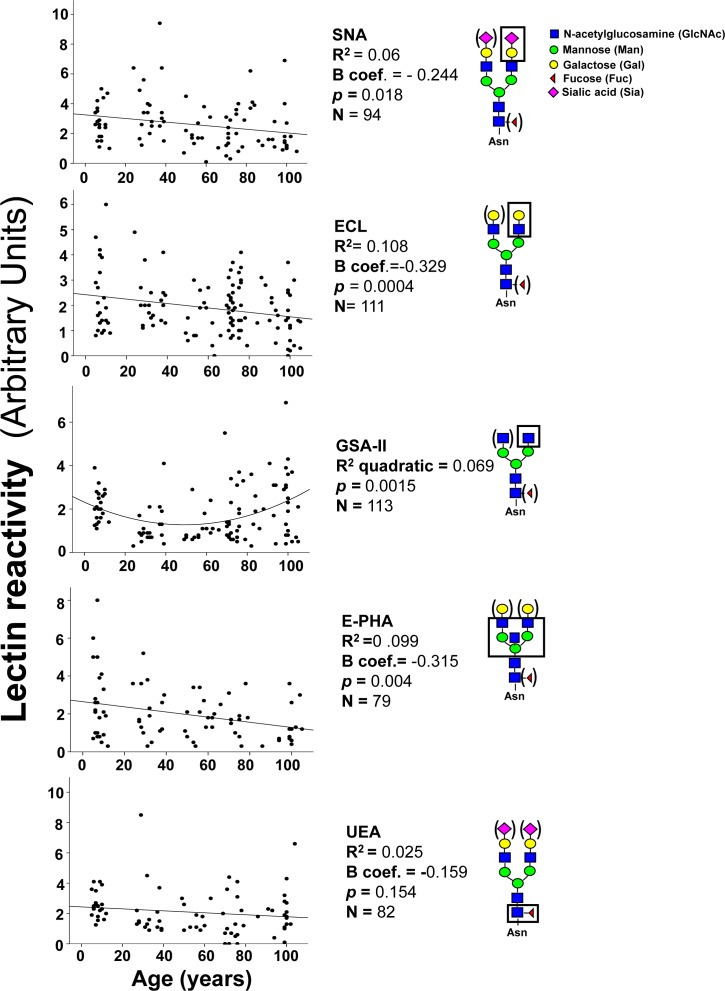
Lectin blot analysis of IgG glycosylation Diluted plasma samples were analyzed after blotting with five lectins. In each gel, the reactivity associated with IgG heavy chains of plasma samples was normalized with that of fixed amounts of commercially available IgG, used as an internal standard. Correlation analysis indicated significant inverse linear relationship with age for SNA, ECL and E-PHA and a positive quadratic relationship for GSA-II. The structures recognized by each lectin is boxed. Structures in parenthesis can be present or absent without affecting recognition by the lectin.

The majority of plasmatic glycoproteins is synthesized by the liver; antibodies represent a remarkable exception, being synthesized by B lymphocytes and plasma cells. Owing to the very high level of ST6GAL1 in hepatocytes, is not surprising that α2,6-sialylation is a major modification of plasmatic glycoproteins. To compare the level of α2,6-sialylation of antibodies with that of other plasmatic glycoproteins, diluted preparations of human plasma were analyzed by lectin blot, using the sialyl α2,6Gal/GalNAc-specific lectin from *Sambucus nigra* (SNA) as a probe [32]. Using undiluted plasma, a very large number of glycoproteins provided a very strong SNA signal, revealing that α2,6-sialylation is a very frequent modification (data not shown). For this reason we used 100 fold diluted plasma samples to identify and characterize only major α2,6-sialylated glycoproteins which were successively isolated as described in Materials and Methods and identified by MALDI-TOF/TOF analysis as thrombospondin-1, IgM heavy chain, fibrinogen β- and γ-chains and IgG heavy chain (Figure 3A). Preliminary analysis of the level of α2,6-sialylation of the above mentioned glycoproteins in a limited group of children, young, old and centenarians was performed to establish which glycoprotein(s) displayed age-associated changes of α2,6-sialylation. As shown in Figure 3B, only the heavy chains of IgG underwent a dramatic and significant age-dependent decrease of α2,6-sialylation. Little or no age-dependent sialylation changes were displayed by glycoproteins of non-lymphocytic origin (thrombospondin-1 and fibrinogen β and γ chains) but also by IgM heavy chains. This indicates that the age-dependent decrease of α2,6-sialylation observed in IgG heavy chains was very specific. Thus, further analysis was focused on IgG heavy chain glycosylation, using lectins specific for carbohydrate determinants known to be present on the Asn_297_ residue. The lectins from *Erythrina cristagalli* (ECL) specific for terminal galactose [33]; *Griffonia simplicifolia* II (GSA-II), specific for terminal GlcNAc [34]; erythroagglutinin from *Phaseolus vulgaris*, specific for bisecting GlcNAc [35] and *Ulex europeus* (UEA), specific for L-fucose [36] were used. An example of the lectin reactivity displayed by plasma samples and commercial IgG is shown in Supplementary Figure 1. The age-dependent variations of lectin reactivity are displayed in Figure 4. SNA reactivity showed a significant (*p* = 0.018 after Bonferroni correction) age-dependent inverse linear relationship. In fact the reactivity progressively declined from infancy to centenarians. ECL reactivity displayed a similar trend with a significant (*p* = 0.0004 after Bonferroni correction) age-dependent reduction. GSA-II displayed a significant quadratic relationship (*p* = 0.0015) with high reactivity in children, decreased reactivity in young adults and a marked age-dependent progressive increase. E-PHA reactivity displayed a significant (*p* = 0.004) linear decrease from children to centenarians, while no significant relationship with age was shown by UEA reactivity (Figure 4).

The reactivity with GSA-II and ECL can be considered surrogates of peak 1 and 6 determined by DSA-FACE, respectively. In Figure S2A, is shown that the Log of the ratio between the reactivity with these two lectins [Log (GSA-II/ECL)] has a very good predictive value of calendar age (*p* < 0.0001). Correlation between the GlycoAge test and the Log(GSA-II/ECL) (Figure 2SB) was also highly significant (*p* < 0.0001).

**Figure 5:**
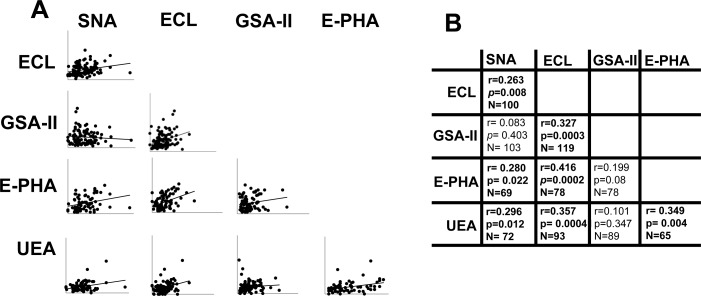
Correlation among lectin reactivities **A.** The reactivity of each lectin was correlated with that of every other lectin evaluated. The significance of each pair is reported in **B.** Statistical significant relationships are indicated in bold.

To ascertain whether the age-associated changes of lectin reactivity were coordinately regulated, we performed correlation analysis between the reactivity of the different lectins (Figure 5). We observed that anti-inflammatory glycotypes, such as α2,6-sialylation (SNA), terminal galactosylation (ECL) and terminal fucosylation (UEA), correlated each other and with bisecting GlcNAc (E-PHA). On the other hand, the pro-inflammatory terminal GlcNAc modification (GSA-II) did not correlate with SNA, UEA and E-PHA reactivity although unexpectedly it correlated with terminal galactosylation (ECL). These data indicate that anti-inflammatory sugar modifications of IgGs are often coordinately regulated.

## DISCUSSION

The present work aims at shedding light in the complex balance of circulating pro-and anti-inflammatory molecules which are at the basis of the age-associated inflammatory status known as inflammaging [37]. Among these molecules, antibodies play a relevant role and provide one of the best examples of how the biological functions of a glycoprotein are regulated by its sugar chains. The glycan linked to Asn_297_ of heavy chains can provide the IgG with pro- or anti-inflammatory roles by modulating the interaction of IgG with different effectors of the innate or adaptive immune system [38, 39]. In particular, IgG-G0 exert pro-inflammatory effects [40-44], while core-fucosylated IgG exert anti-inflammatory effects by reducing ADCC [45, 46]. α2,6-sialylated IgG have been reported to be the main responsible of the powerful transient anti-inflammatory effect induced by the infusion of high doses human IgG (IVIG) [47, 48]. However, on the importance of α2,6-sialylation of IgG in IVIG conflicting results have been published [49-55]. Neither the origin of IgG-G0, nor that of α2,6-sialylated IgG [56, 57] has been unambiguously explained by the level of expression of the cognate glycosyltransferases in B-lymphocytes. Thus, the possibility of a pivotal role played by extracellular glycosylation, as demonstrated in mice for plasmatic ST6GAL1 [31, 58, 59] is intriguing.

Our results show for the first time that plasmatic B4GALTs activity exhibits a linear increase from infancy to extremely old age, while plasmatic ST6GAL1 activity is present at similar levels during adulthood, but is expressed at a high level only in infancy, old subjects and centenarians. However, neither enzyme showed a positive correlation with their cognate sugar structures on IgG (B4GALTs/galactosylation; ST6GAL1/α2,6-sialylation), strongly suggesting that extracellular glycosylation does not play a major role in determining the human antibody glycotype. The lost after age adjustment of the positive association between plasmatic B4GALTs and the GlycoAge test strongly suggests that the two parameters reflect different physio-pathological mechanisms. On the contrary, the positive relationship between plasmatic ST6GAL1 and the GlycoAge test, which was conserved after age-adjustment, suggests that the two parameters share a common physio-pathological mechanisms.

In rodents, ST6GAL1 behaves as an acute phase protein [60, 61] and plays a role in down-regulating inflammation. In fact, mice knock out for the hepatic ST6GAL1 promoter display a low levels of plasmatic ST6GAL1 and an exaggerated neutrophil accumulation in response to inflammatory stimuli [59]. It has been shown in mice that the extracellular sialylation of hematopoietic stem cells by plasmatic ST6GAL1 limits neutrophil production [58, 62, 63]. Altogether, these data are consistent with a model in which the inflammation-dependent elevation of plasmatic ST6GAL1 has the effect of limiting the extent of the inflammatory response. Translating the data from experimentally inflamed mice to old humans, it can be hypothesized that the elevation of plasmatic ST6GAL1 is not a bad consequence of inflammaging but rather an attempt of the body to limit its adverse effects. This view is supported by the observation that the offspring of centenarians, who are at lower risk of inflammatory age-related diseases, have plasmatic ST6GAL1 activity higher than NLPO. Moreover, only in NLPO ST6GAL1 activity showed a positive association with some inflammatory markers, suggesting that the genetic background of OC can help ST6GAL1 to keep low the level of inflammation. The lack of a positive relationship between markers of liver damage and plasmatic B4GALTs or ST6GAL1 activities suggests that their level was not due to liver damage. Rather, it could be the result of an active proteolytic process which, for ST6GAL1, has been shown to be mediated by enzymes such as BACE1 [64-66].

The lectin blot approach we used, albeit limited by a lower throughput compared with DSA-FACE or mass spectrometry, offers the advantage of detecting glycosylation changes associated with specific glycoproteins and provides a clear cut answer to the presence/absence of a specific carbohydrate structure on IgGs heavy chains. Through this approach we documented changes of IgG glycosylation consistent with previous literature [12-16, 67-71]. The significant associations we observed between the reactivity with UEA, SNA and ECL, three lectins detecting sugar chains associated with an anti-inflammatory phenotype of IgGs, suggests the possibility of a coordinate regulation of glycosylation driving the inflammatory properties of IgG.

In conclusion, in this study we have identified a new marker of calendar age, plasmatic B4GALTs and revealed a more complex pattern of age-dependence of plasmatic ST6GAL1, suggestive of a possible role in limiting the effects of inflammaging. We have also shown that in humans, the glycosylation of antibodies is poorly affected by plasmatic glycosyltransferases, while in aging it undergoes a shift toward an inflammatory glycotype.

## MATERIALS AND METHODS

### Plasma samples

We studied 125 subjects of six different age groups: 5-12 years (*N* = 23, mean age ±SD 7.6±1.95; M/F = 17/6), 24-39 (*N* = 20, mean age 32.1±4.3; M/F = 10/10), 40-60 (*N* = 13, mean age 53.3±5.3; M/F = 6/7), 61-80 (*N* = 29, mean age 71.63±4.2; M/F = 14/15), 81-99 (*N* = 20, mean age 88.9±5.0; M/F = 11/9), 100-105 (*N* = 20, mean age 100.4±2.0; M/F = 10/10). Additionally, a cohort of 19 offspring of centenarians (OC) (mean age 72.9 ± 2.5; M/F = 10/9) was recruited and compared with 19 sex- and age-matched non-long lived parents offspring (NLPO). These individuals, (mean age 72.3 ± 2.5; M/F = 9/10) are included in the 61-80” group. NLPO have both parents born in the same birth cohort of centenarians (that is 1900-1908) but dead before the threshold age over which subjects were classified as long-lived as previously described [72]. Overnight fasting plasma samples were obtained in the morning and within 2 hours from venipuncture by centrifugation at 2000 x *g* for 20 min at 4°C, rapidly frozen and stored at −80°C. Subjects enrolled for evaluation of inflammatory status no used medication that could influence the inflammatory markers, such as steroid or nonsteroidal drugs and immunomodulatory agents, during the week before the blood drawn. Investigation has been conducted in accordance with the ethical standards and according to the Declaration of Helsinki and according to national and international guidelines and has been approved by the Ethical Committee of Sant'Orsola-Malpighi University Hospital (Bologna, Italy). Written consent was obtained from all subjects. For children, written consent was obtained from parents and also from children through pictures and simplified information.

### Plasmatic glycosyltransferase activities

β-galactoside α2,6-sialyltransferase (ST6GAL1) activity was measured in plasma specimens, devoid of any type of blood cell, using asialotransferrin as an acceptor, essentially as previously described [73, 74]. The reaction mixture contained in a final volume of 50 μl : 0.08 M Na-cacodylate buffer pH 6.5, 5 μM unlabeled CMP-sialic acid and 1μl (0,001 mCi/ml) of CMP-[^3^H] sialic acid, 500 μg asialotransferrin as acceptor glycoprotein and 5 μl of human plasma. After 3 hours at 37°C the acid-insoluble radioactivity was determined as previously described [73, 74].

The reaction mixture for the determination of B4GALTs activity contained in a final volume of 20.5 μl, 0.1 M Tris/HCl pH 8, 9.75 mM MnCl_2_, 4.9 mM CDP-choline, 1 μl of UDP-[^14^C]Galactose (0.025 mCi/ml), 0.4 mM ATP, 5 μl of plasma and 550 μg ovalbumin. After 3 hs at 37°C, the acid insoluble radioactivity was measured as described above.

### Lectin blot analysis

Ten microliters of plasma diluted 100 fold with waterBS were electrophoresed in reducing conditions according to Laemmli protocol [75]. The following lectins (Sigma) were labelled with digoxigenin (Roche) as indicated by the provider and used for the detection of the indicated sugar chains: *Sambucus nigra* (SNA) for α2,6-linked sialic acid [32]; *Erythrina cristagalli* (ECL) for terminal galactose [33]; *Griffonia simplicifolia* II (GSA-II), for terminal N-acetylglucosamine (GlcNAc) [34]; erythroagglutinin from *Phaseolus vulgaris*, for bisecting GlcNAc [35] and *Ulex europeus*, for L-fucose [36]. Blotting and detection were performed as previously described [76].

### SNA-agarose precipitation

Four hundred microliters of plasma were brought to a final volume of 0.5 ml in PBS, containing 1% NP40, 1% deoxycholic acid, and protease inhibitors. Then, 20 μl of 2 mg/ml SNA-biotin (Vector Laboratories, Burlingame, CA, USA) were added and the samples were stirred for 5 h at 4°C. Fifty microliters of streptavidin-agarose (Vector Laboratories) were then added and the samples were stirred overnight at 4°C. Samples were then centrifuged in a minifuge and washed three times with PBS containing 1% NP40, 1% deoxycholic acid, and protease inhibitors and once with 50 mM Tris-HCl, 15 mM NaCl. After centrifugation, samples were resuspended with reducing sample buffer, incubated at 60°C 15 minutes and electrophoresed.

### GlycoAge test

N-glycans analysis was performed by DNA Sequencer-Aided, Fluorophore-Assisted Carbohydrate Electrophoresis (DSA-FACE) as described elsewhere [17]. Briefly, 5 μl of plasma were incubated with 2 μl of a denaturing buffer (5% SDS in 10 mM NH_4_HCO_3_) in a 96 well plate, sealed and incubated for 5 min at 95°C. Enzymatic N-glycan release from the protein fraction was performed by adding 3 μL of a release mixture containing 3.33% NP-40 and 33 Units of recombinant PNGase F (New England Biolabs) in 10 mM NH4HCO3, pH 8.3 and incubating for 3 h at 37°C. The released plasma N-glycans were then desialylated with 2 mU of Neuraminidase of *Arthrobacter ureafaciens* (Roche) in 5mM of NH4Ac, pH 5. 2 μl of the desialylated N-glycans were dried completely into a new plate, at 60°C for 1 h; after that, 2 μL of fluorophore 8-amino-1,3,6-pyrenetrisulfonic acid (APTS) labeling buffer (1:1 mixture of 20 mM APTS in 1.2 M citric acid and 1 M NaCNBH3 in DMSO) were added per sample. The reaction was stopped by adding 150 μL of mQ water to each well. 10 μL of APTS labeled and desialylated glycans were analyzed using a CE-based ABIA sequencer (Applied Biosystems). The data are processed by using Peak Scanner software (Applied Biosystem). We measured the intensities (heights) of 10 N-glycans peaks and then normalized to the sum of the heights of all peaks. The GlycoAgeTest is evaluated by calculating the log of the ratio of the concentration of two diantennary N-glycans, both core fucosylated: an agalactosylated (NGA2F, or peak 1) and a digalactosylated (NA2F, or peak 6) [15].

### Statistical analysis

The relationship between age and plasmatic glycosyltransferenses and the GlycoAge test was assessed by a linear regression analysis using age as independent variable, and each single variable as dependent variable. We performed the Kruskal-Wallis not parametric test to compare the different age-ranges and we reported the *p*-values calculated after Bonferroni correction for multiple comparisons.

In order to test the relationship between the GlycoAge test and either plasmatic glycosyltransferase, independently of the age effect, we regressed each parameter (GlycoAge test, plasmatic B4GALTs and ST6GAL1) on age of subjects. Subsequently, the unstandardized residuals of each of these age-adjusted parameters (GlycoAge test, plasmatic B4GALTs and ST6GAL1) were used for further linear regression analysis among these parameters (between GlycoAge test and plasmatic B4GALTs, between GlycoAge test and plasmatic ST6GAL1, as well as between ST6GAL1 and B4GALTs).

Correlation among plasmatic glycosyltransferases and inflammatory and liver damage markers were analyzed in centenarians, their offspring and non-long lived parents offspring and were presented as Spearman correlation coefficients. A two-tailed *p*-value of < 0.05 was considered significant. All analyses were carried out using SPSS for Machintosh, version 20.

### MALDI/TOF-TOF identification of SNA-reactive glycoproteins

The selected protein bands were excised from the gel and processed for MALDI MS analysis following an already published procedure [77]. Briefly, the protein gel bands were washed, destained, reduced with DTT, alkylated with IAA, “in gel” digested with trypsin (Promega, Madison WI, USA) and the resulting peptides were extracted from the gel. Protein digests were desalted, concentrated and spotted onto a MALDI plate using ZipTips (Millipore, Billerica, MA, USA) following the manufacturer's instructions. For the matrix preparation, a solution of 7-8 mg/ml α-cyano-4-hydroxycinnamic acid in 50% ACN/0.1% TFA was used. Samples were analyzed using a 4700 Proteomics Analyzer MALDI-TOF/TOF (AB SCIEX, Framingham, MA, USA). Peptide mass fingerprint (PMF) data were collected in positive MS reflector mode for the mass window m/z 700-4000 using trypsin autolysis peaks for internal calibration. Some of the highest intensity non-tryptic peaks were selected for MS/MS analysis. The MS and MS/MS spectra were analyzed using the software GPS Explorer (Version 3.6, AB SCIEX), and were searched together against the UniProtKB protein sequence database for the Homo sapiens taxonomic selection using the Mascot protein search engine (Version 2.1.04, Matrix Science, UK). The search included peaks with a signal-to-noise ratio greater than 10, allowed for up to two missed trypsin cleavage sites. A fixed modification of cysteine (carbamidomethylation) and a variable modification of methionine (oxidation) were used in the search parameters. To be considered a match a confidence interval (CI), calculated by the AB SCIEX GPS Explorer software, of at least 99% was required.

### Determination of inflammatory and liver damage markers

Serum levels of glutamic-oxaloacetic transaminase (GOT), glutamic-pyruvic transaminase (GPT), gamma glutamyl transferase (GGT), C reactive protein (CRP), von Willebrandt Factor (vWF) were measured by standard biochemical methods in the clinical laboratory of S. Orsola Hospital, Bologna, Italy. Plasmatic levels of resistin, interleukine-6 (IL-6), interleukine-10 (IL-10), tumor necrosis factor-α (TNF-α) and amyloid A (SAA) were measured by multiplex sandwich ELISA technology (SearchLight, Aushon Biosystems, Billerica, MA) according to the manufacturer's instructions and described previously [78].

## SUPPLEMENTARY MATERIAL FIGURES


