# Supplementary material for: Identification of novel plasma glycosylation-associated markers of aging
Source: Oncotarget. 2016 Jan 28;7(7):7455–68. doi: 10.18632/oncotarget.7059 (PMC4884931; doi:10.18632/oncotarget.7059)
Supplement: Supplementary file 1 [file oncotarget-07-7455-s001.pdf]

Identification of novel plasma glycosylation-associated markers of aging

Supplementary Material

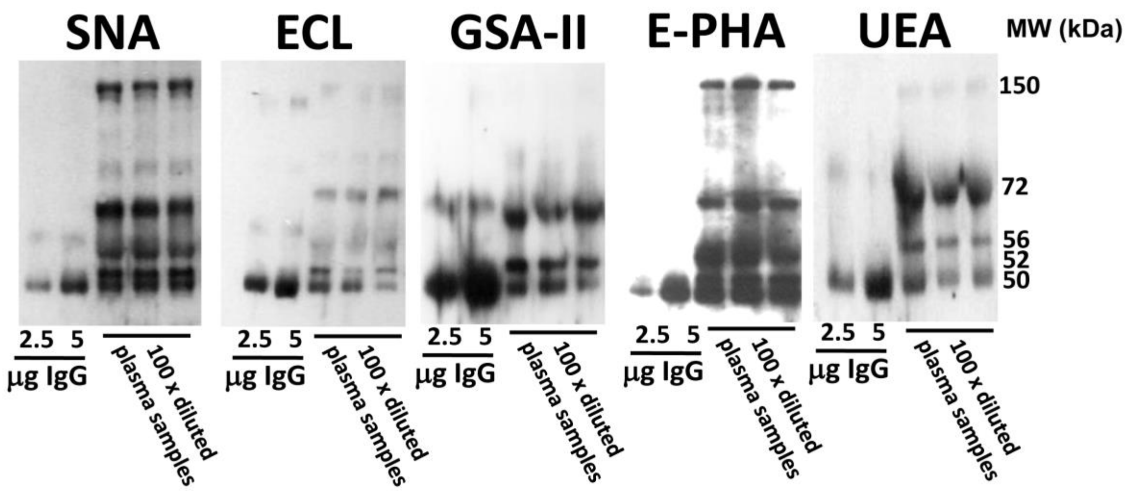

Supplementary Fig. 1. Example of lectin blot analysis of plasma samples. In the first two lanes 2.5 and 5 micrograms of commercial IgG were loaded as standard. Ten microliters of plasma diluted 100 fold with water were analyzed. Dilution of plasma was necessary to avoid overload of the gel. The plasma specimens analyzed in these five blots were not the same.

A: The Log of the ratio between the reactivity of GSA-II and ECL [Log (GSA-II/ECL)] can be considered a surrogate of the GlycoAge test. Log (GSA-II/ECL) showed a highly significant linear relationship with age.

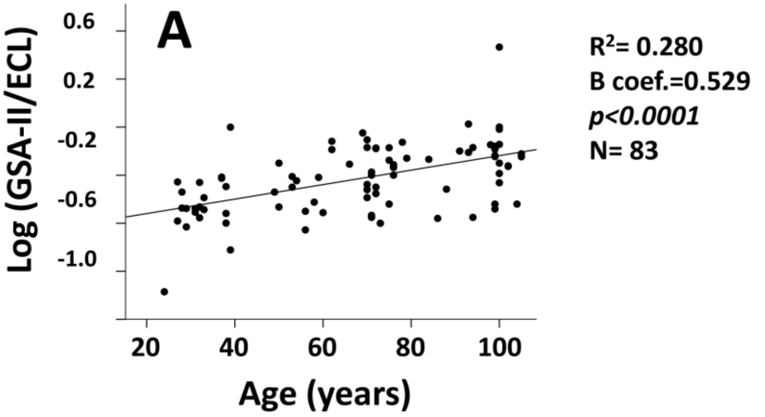

B: Comparison between the Log (GSA-II/ECL) and the GlycoAge test revealed a highly significant linear relationship.

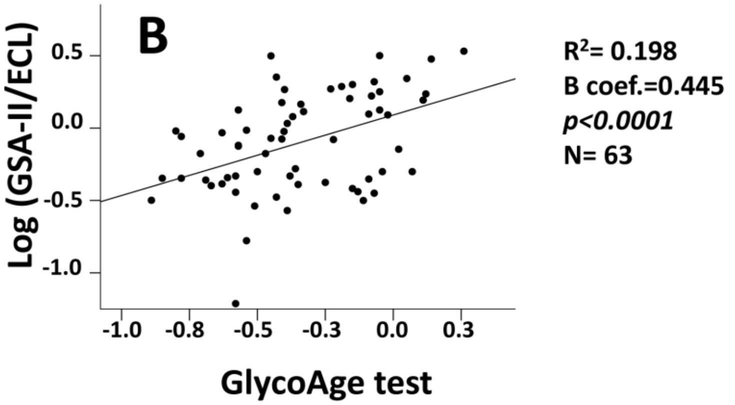

Supplementary Figure 2:
